# Preoperative Grading of Rectal Cancer with Multiple DWI Models, DWI-Derived Biological Markers, and Machine Learning Classifiers

**DOI:** 10.3390/bioengineering10111298

**Published:** 2023-11-09

**Authors:** Mengyu Song, Qi Wang, Hui Feng, Lijia Wang, Yunfei Zhang, Hui Liu

**Affiliations:** 1 Department of Radiology, Fourth Hospital of Hebei Medical University, No.12 Jiankang Road, Shijiazhuang 050000, China; 2 Central Research Institute, United Imaging Healthcare, Shanghai 201800, China

**Keywords:** diffusion weighted MRI, machine learning, rectal neoplasms, intravoxel incoherent motions, diffusion kurtosis imaging

## Abstract

**Background:** this study aimed to utilize various diffusion-weighted imaging (DWI) techniques, including mono-exponential DWI, intravoxel incoherent motion (IVIM), and diffusion kurtosis imaging (DKI), for the preoperative grading of rectal cancer. **Methods:** 85 patients with rectal cancer were enrolled in this study. Mann–Whitney U tests or independent Student’s *t*-tests were conducted to identify DWI-derived parameters that exhibited significant differences. Spearman or Pearson correlation tests were performed to assess the relationships among different DWI-derived biological markers. Subsequently, four machine learning classifier-based models were trained using various DWI-derived parameters as input features. Finally, diagnostic performance was evaluated using ROC analysis with 5-fold cross-validation. **Results:** With the exception of the pseudo-diffusion coefficient (D_p_), IVIM-derived and DKI-derived parameters all demonstrated significant differences between low-grade and high-grade rectal cancer. The logistic regression-based machine learning classifier yielded the most favorable diagnostic efficacy (AUC: 0.902, 95% Confidence Interval: 0.754–1.000; Specificity: 0.856; Sensitivity: 0.925; Youden Index: 0.781). **Conclusions:** utilizing multiple DWI-derived biological markers in conjunction with a strategy employing multiple machine learning classifiers proves valuable for the noninvasive grading of rectal cancer.

## 1. Introduction

Rectal cancer is a leading cause of human death. The histopathological grade is an important prognostic factor of rectal cancer [1,2]. High-grade rectal cancer always signifies the poor prognosis, including the high probability of both recurrence and metastasis [3,4]. Additionally, previous research indicates that patients with high-grade rectal cancer always have a much shorter median survival time [5,6]. Furthermore, preoperative radiotherapy and chemotherapy are often required for patients with high-grade rectal cancer. At present, histopathological examination serves as the gold standard for evaluating the grade of rectal cancer during clinical practice. Nevertheless, the invasiveness, potential sampling bias, and time-consumption are the insurmountable limitations of histopathological examination [7,8,9]. Therefore, non-invasive, cost-effective, and convenient approaches are urgently needed for grading rectal cancer.

As a non-invasive and cost-effective imaging technique, diffusion weighted magnetic resonance imaging (DW-MRI) has shown tremendous clinical potential for cancer diagnosing, and cancer grading, as well as therapeutic response prediction [10,11]. Moreover, numerous DWI models, including the conventional DWI (mono-exponential DWI), IVIM (intravoxel incoherent motions), DKI (diffusion kurtosis imaging), and so on, have been proposed for characterizing tumor from different perspectives [12,13,14,15,16,17,18,19,20,21]. However, the biological insights provided by a lot of DWI-derived parameters mainly include cellularity, vascularity and micro-structural heterogeneity [12]. For instance, the conventional DWI-derived apparent diffusion coefficient (ADC) is able to provide the characterization of cellular density. Differently, IVIM-derived perfusion fraction (f) and so on, together with the DKI-derived apparent kurtosis coefficient (K_app_) and so on, are able to characterize the vascularity and micro-structural heterogeneity, respectively. Therefore, integrating these biological markers together will benefit the clinical application via more comprehensively characterizing the tumor from multiple perspectives.

Several researchers have explored the utility of DWI techniques for assessing rectal cancer. For instance, studies employing mono-exponential DWI, IVIM, DKI, and RSI have demonstrated potential in noninvasively predicting the grade of rectal cancer [15,19,22,23]. Nonetheless, there have been limited efforts to utilize diverse DWI biomarkers to evaluate rectal cancer. Additionally, there is a scarcity of reports on the integration of various DWI biomarkers using machine learning-based models to comprehensively characterize rectal neoplasms.

**Figure 1:**
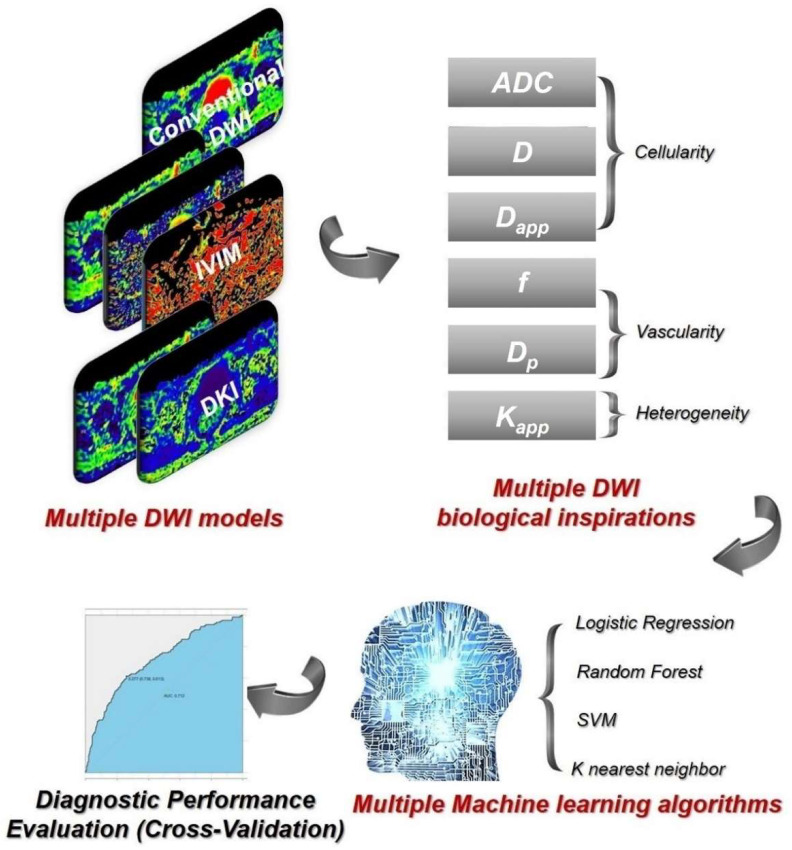
Technique flowchart of grading rectal cancer with multiple DWI models and multiple DWI biological markers, along with multiple machine learning classifiers.

In this study, we formulated the following hypotheses: the integration of mono-exponential DWI, IVIM, and DKI techniques would be sufficient to elucidate three key DWI-related biological aspects: cellular density, perfusion associated with vascularity, and structural heterogeneity. The amalgamation of these DWI-derived biological insights could enhance the comprehensive characterization of tumors from various perspectives, thus holding significant potential for the accurate grading of rectal cancer. Machine learning, as a robust technique, could effectively incorporate diverse quantitative biomarkers by utilizing them as input features. Consequently, the primary aim of this research is to preoperatively grade rectal cancer using multiple DWI models, multiple DWI-derived biological parameters, and various machine learning classifiers. The technique flowchart is illustrated in Figure 1.

## 2. Materials and Methods

### 2.1. Patients

Ethical approval was obtained from the local Institute Review Board (IRB) under Approval Number #MEC089, and written informed consent was obtained from each patient. A total of 105 patients were enrolled in this prospective study between May 2020 and April 2021, following the established inclusion and exclusion criteria, which were based on previous research as a reference [7]. The inclusion criteria were: (1) patients with a strong suspicion of rectal cancer according to other imaging examinations or their medical history, as well as those with confirmed rectal cancer through endoscopic biopsy. The exclusion criteria: (1) Patients who received any adjuvant treatment between the MR examination and subsequent surgical treatment. (2) Patients with a significant time gap (≥10 days) between the MR examination and the subsequent surgical procedure. (3) Patients without a surgical treatment history and records at our institute, leading to a lack of histopathological results. (4) Patients with poor image quality of DWI images due to artifacts or other factors. (5) Patients with lesions that were too small to be identified in DWI images.

In this prospective study, we initially recruited 105 patients. However, we excluded 7 patients due to pre-operative neoadjuvant therapy, 2 patients due to poor MR image quality, 6 patients because the time interval between MR examination and surgery exceeded ten days, and 5 patients due to missing histopathologic grade results. Ultimately, our study comprised 85 participants, consisting of 45 men and 40 women. This cohort included 17 patients classified as WHO-Grade 1 (G1), 36 patients as WHO-Grade 2 (G2), and 32 patients as Grade 3 (G3) according to the WHO classification.

### 2.2. MRI Examinations

All the MRI measurements were performed with a 3.0 T scanner (uMR 780, United Imaging Healthcare, Shanghai, China) and a twelve-channel coil. The MRI protocols contained a T2-weighted Fast Spin Echo sequence termed as FSE T2WI (repetition time (TR): 4200 ms; echo time (TE): 103.1 ms; flip angle (FA): 110°; matrix: 336 × 432; field of view (FOV): 280 × 360 mm^2^; slice thickness: 6 mm), T1-weighted Fast Spin Echo sequence termed as FSE T1WI (TR: 649.0 ms; TE: 10.7 ms; FA: 110°; matrix: 480 × 570; FOV: 320 × 380 mm^2^; slice thickness: 6 mm), Dynamic three-dimensional T1 weighted gradient echo (GRE) sequence (TR: 3.3 ms; TE: 1.45 ms; FA: 10°; matrix: 336 × 480; FOV: 280 × 499 mm^2^) and single shot-spin echo-echo planar imaging sequence termed as SS-EPI (TR: 4800 ms; TE: 88 ms; FA: 90°; section thickness: 4 mm; matrix: 95 × 112; FOV: 180 × 240 mm^2^; voxel size: 2.52 × 2.14 × 4 mm^3^; b values: 0, 10, 20, 30, 50, 80, 100, 150, 200, 400, 600, 800, 1500 and 2000 s/mm^2^; scanning time: 5 min and 14 s).

Furthermore, to mitigate the adverse effects of intra-rectal air on reliable DWI examinations, patients were instructed to maintain steady and smooth breathing, while efforts were made to prevent any irregular inhalations. It is important to emphasize that both DKI and IVIM analyses were conducted based on the SS-SE-EPI sequence mentioned earlier, with varying selections of b values for subsequent post-processing.

### 2.3. Image Analysis

#### 2.3.1. DWI Parametric Maps

All original data underwent processing using a custom in-house prototype software developed in MATLAB (MathWorks, Natick, MA, USA). For mono-exponential diffusion-weighted imaging (DWI) analysis, the fitting algorithm employed in accordance with prior research can be expressed as follows: $S_{b}/S_{0}=\exp\left(-bADC\right)$ where $S_{b}$ is the signal intensity when specific b values (800 s/mm^2^) are applied, $S_{0}$ is the DWI signal intensity when b value of 0 is applied, and ADC is the abbreviation of apparent diffusion coefficient. For the IVIM DWI model, the fitting algorithm was according to previous research [24], and can be expressed as: $S_{b}/S_{0}=\left(1-f\right)\exp\left(-bD\right)+fexp(-bD_{p})$, where $S_{b}$ is the signal intensity when specific b values are applied, $S_{0}$ is the DWI signal intensity when b value of 0 is applied, f is the abbreviation of perfusion fraction representing the proportion of protos related with “pseudo diffusion” (micro-circulation), D is defined as the abbreviation of diffusion coefficient representing the pure diffusivity, and $D_{p}$ is defined as the abbreviation of pseudo diffusion coefficient representing the incoherent microcirculation. The quantitative pixel-wise parameters and parametric maps can be generated with the b values including 0, 10, 20, 30, 50, 80, 100, 150, 200, 400, 600, and 800 s/mm^2^ as the input data on a voxel-by-voxel basis. For DKI DWI model, the fitting algorithm was according to previous research [25], and can be expressed as: $\ln\left(S_{b}\right)=\ln\left(S_{0}\right)-b\cdot D_{app}+1/6\cdot b^{2}{D_{app}}^{2}K_{app}$, where $S_{b}$ and $S_{0}$ are similar to those of IVIM, $D_{app}$ is the abbreviation of apparent diffusion coefficient representing the apparent diffusivity, $K_{app}$ is defined as the abbreviation of apparent kurtosis coefficient representing the deviation from a perfect Gaussian distribution. The quantitative pixel-wise parameters and parametric maps can be generated with the b values including 0, 800, 1500, and 2000 s/mm^2^ as the input data on a voxel-by-voxel basis. We used the Levenberg–Marquardt non-linear fitting algorithm for parametric fitting in this study. The fitting bounds for D, D_p_, f, D_app_, K_app_ were as follows: 0.0 to 5.0 × 10^−3^ mm^2^/s, 0.0 to 80.0 × 10^−3^ mm^2^/s, 0.0 to 0.8, 0.0 to 5.0 × 10^−3^ mm^2^/s, 0.0 to 2.5, respectively.

#### 2.3.2. Definition of Volume of Interests (VOIs)

Following the methodology established in previous studies [7,8], three experienced radiologists, each having significant expertise in the field (Q.W with 32 years of experience, H.L with 22 years, and MY.S with 5 years), conducted an independent review of the MRI images. These radiologists were blinded to the pathological results and delineated freehand volume of interests (VOIs), along the boundaries of the low-signal regions of the tumor on the ADC, D, and D_app_ maps. This meticulous approach aimed to ensure maximum inclusion of the entire tumor within the VOI while excluding areas of necrosis, cysts, and hemorrhage. Subsequently, these VOIs were automatically replicated onto other parametric maps, including the D_p_ map, f map derived from the D map, and K_app_ map derived from the D_app_ map.

To calculate the mean values of the DWI-derived parameters, we employed a whole-lesion averaging approach. This approach ensured that the measurements were made across the entirety of the tumor region. To enhance the reliability of our analysis, we averaged the measurements of DWI parameters obtained by the three different observers. For making measurements and defining the volume of interests, we utilized an open-source software application designed for 3D medical image segmentation, known as ITK-SNAP (http://www.itksnap.org/pmwiki/pmwiki.php, accessed on 10 August 2021).

### 2.4. Histopathological Evaluation

The pathological evaluations were conducted by experienced pathologists, each possessing more than 5 years of expertise, utilizing hematoxylin–eosin (H&E) stained surgical specimens. Histological grading was performed in accordance with the WHO grading criteria [26]. Patients were categorized into one of three grades: grade 1 (WHO-G1), grade 2 (WHO-G2), or grade 3 (WHO-G3) based on the presence of gland-like structures. Specifically: WHO-G1: patients with more than 95% gland-like structures. WHO-G2: patients with gland-like structures ranging from 50% to 95%. WHO-G3: patients with less than 50% gland-like structures. Furthermore, WHO-G1 and WHO-G2 rectal cancers were defined as low-grade rectal cancer, while WHO-G3 was defined as high-grade rectal cancer.

### 2.5. Statistical Analysis

The inter-observer agreement and reproducibility were assessed using the intra-class coefficient (ICC). Inter-observer agreement was categorized as follows: excellent for ICC values between 0.8 and 1.0, substantial for ICC values between 0.6 and 0.8, moderate for ICC values between 0.4 and 0.6, fair for ICC values between 0.2 and 0.4, and poor for ICC values between 0.0 and 0.2. To evaluate the normal distribution of the parameters, the Shapiro–Wilk test was employed. Based on the results of the Shapiro–Wilk test, independent Student’s *t*-tests or Mann–Whitney U tests were used to determine whether significant differences existed between patients with low-grade rectal cancer and those with high-grade rectal cancer. Pearson or Spearman correlation tests were conducted to assess correlations among different DWI-derived parameters, with the correlation coefficient abbreviated as “r.” Subsequently, incorporating IVIM-derived or DKI-derived parameters that exhibited significant differences between low-grade and high-grade patients, machine learning-based diagnostic models, including logistic regression (LG), K-nearest neighbor (KNN), support vector machine (SVM), and random forest (RF), were sequentially established. LG, KNN, SVM, and RF are all machine learning classifiers that take quantitative features as input and provide probabilities for various categories as output. LG is a linear model used for binary classification. It estimates the probability of an instance belonging to a class. KNN is a non-parametric algorithm that classifies based on the majority class of its closest neighbors in feature space. SVM finds the optimal hyperplane maximizing class separation, suitable for linear and non-linear classification. RF is an ensemble method that combines decision trees to enhance classification accuracy, reducing overfitting. A receiver operating characteristic curve (ROC) analysis was performed to evaluate the diagnostic performance of individual DWI-derived parameters and the four machine learning models. The assessment included quantitative indices such as the area under the curve (AUC), specificity, sensitivity, and Youden Index. To mitigate potential statistical bias, a 5-fold cross-validation approach was employed to evaluate diagnostic performance. The DeLong test was conducted to compare the AUCs of different models. Statistical analyses were carried out using SPSS software (PASW Statistics 25.0, SPSS Inc., Chicago, IL, USA), Medcalc (MedCalc 9.0.2, Mariakerke, Belgium), R version 3.6.1 (R Core Development Team), and RStudio (RStudio Inc., Boston, MA, USA). R packages, including random forest, e1071, kknn, and caret, were utilized to establish the machine learning models. Statistical significance was determined when the *p*-value was less than 0.05.

## 3. Results

### 3.1. Clinicopathological Characteristics and MR Images

**Figure 2:**
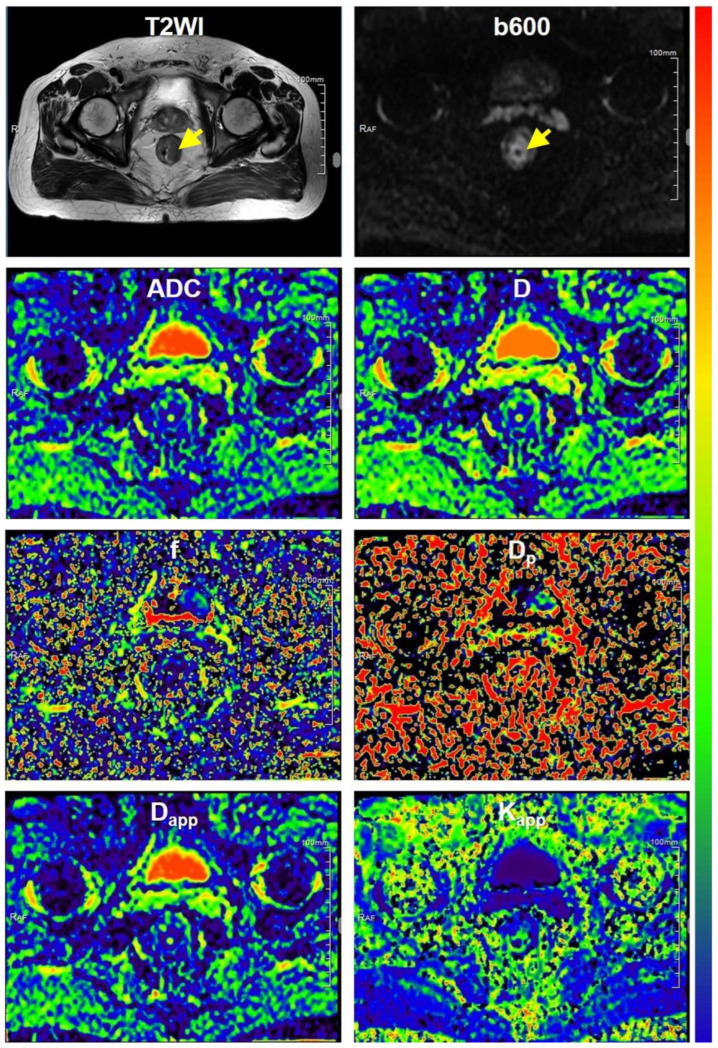
Representative MR images of a patient with low grade rectal cancer. Notes: All parametric maps employed a consistent color bar, spanning from blue to red, to signify the minimum and maximum values, respectively. The parametric ranges for ADC, D D_p_, f, D_app_, K_app_ are as follows: 0.0 to 5.0 × 10^−3^ mm^2^/s, 0.0 to 5.0 × 10^−3^ mm^2^/s, 0.0 to 80.0 × 10^−3^ mm^2^/s, 0.0 to 0.8, 0.0 to 5.0 × 10^−3^ mm^2^/s, 0.0 to 2.5, respectively. The yellow arrows indicate the tumors.

**Figure 3:**
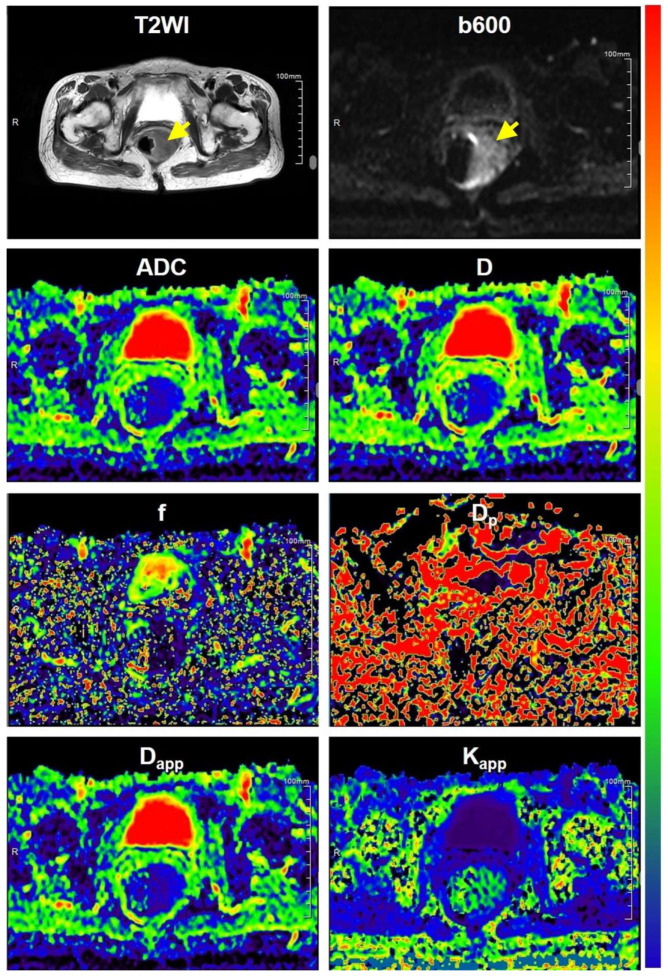
Representative MR images of a patient with high grade rectal cancer. Notes: All parametric maps employed a consistent color bar, spanning from blue to red, to signify the minimum and maximum values, respectively. The parametric ranges for ADC, D D_p_, f, D_app_, K_app_ are as follows: 0.0 to 5.0 × 10^−3^ mm^2^/s, 0.0 to 5.0 × 10^−3^ mm^2^/s, 0.0 to 80.0 × 10^−3^ mm^2^/s, 0.0 to 0.8, 0.0 to 5.0 × 10^−3^ mm^2^/s, 0.0 to 2.5, respectively. The yellow arrows indicate the tumors.

**Figure 4:**
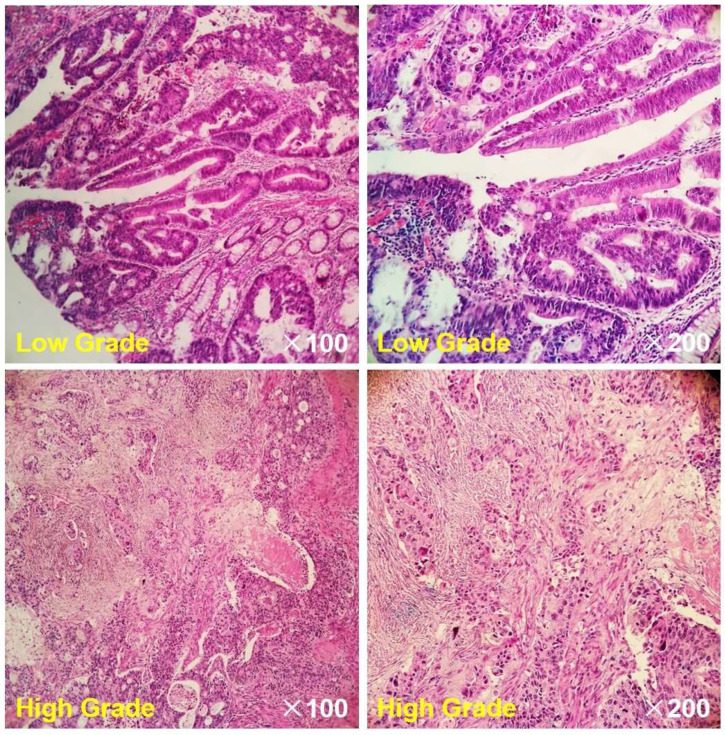
Hematoxylin and Eosin (H&E) stained histopathological sections of a patient with low-grade rectal cancer whose MR images are displayed in Figure 2 and a patient with high-grade rectal cancer whose MR images are displayed in Figure 3.

The baseline clinicopathological characteristics are presented in Table 1. As depicted in Table 1, there were no significant differences observed in terms of age, gender, tumor size (longest diameter), pN stage, expression of Carbohydrate antigen 199 (CA199), or expression of carcinoembryonic antigen (CEA) between low-grade and high-grade rectal cancers (*p* values ranging from 0.085 to 0.703). However, a significant correlation was noted between pT stage and grade (*p* = 0.047). Representative MR images of a low-grade rectal cancer and a high-grade rectal cancer are displayed in Figure 2 and Figure 3, respectively. In Figure 4, histopathologic photographs of two patients, whose MR images are shown in Figure 2 and Figure 3, respectively, are presented. Histopathological examinations revealed that, in comparison to low-grade rectal cancers, high-grade rectal cancers exhibited elevated cellular density with an increased ratio of nuclear to cytoplasm. Additionally, the cell shape, cell size, and other structural characteristics within different tumor regions in high-grade rectal cancer displayed significant variation, implying a more complex microstructure in high-grade rectal cancer.

### 3.2. DWI-Derived Parameters in Different Subgroups

**Figure 5:**
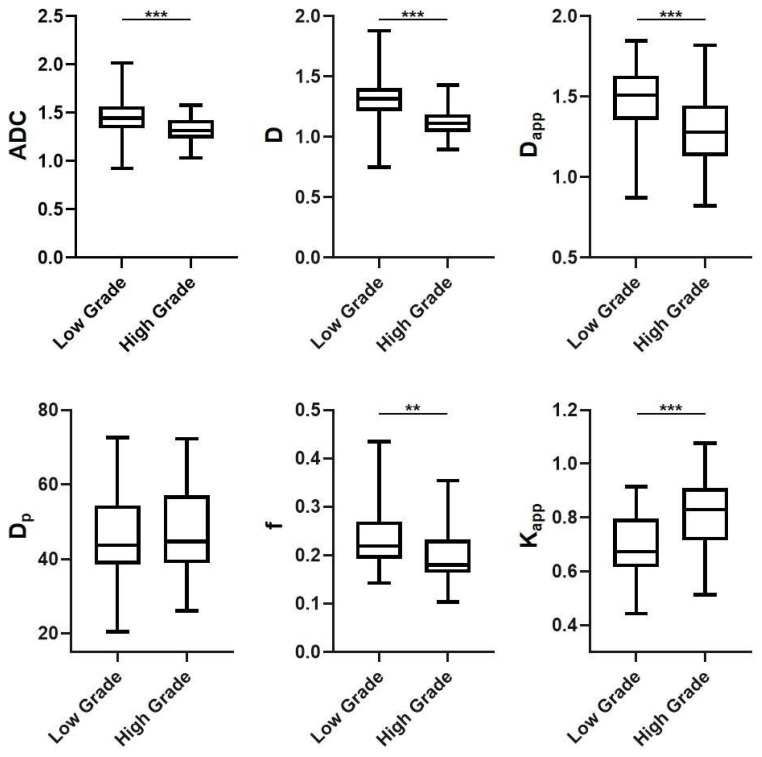
Box-plots of mono-exponential DWI-derived, IVIM-derived and DKI-derived parameters in low grade and high grade rectal cancer. Note: (1) Unit: ADC value: ×10^−3^ mm^2^/s; D value: ×10^−3^ mm^2^/s; D_p_ value: ×10^−3^ mm^2^/s; f value, unitless; D_app_ value: ×10^−3^ mm^2^/s and K_app_, unitless. (2) ** signifies *p* values of less than 0.01 and *** signifies *p* values of less than 0.001.

The inter-observer agreement and reproducibility were quantified using the ICC. The ICCs for various DWI-derived parameters, as measured by three experienced radiologists, fell within the range of 0.751 to 0.862, indicating substantial to excellent inter-observer agreement and reproducibility (Table 2). As depicted in the box plots in Figure 5, the ADC value exhibited a significant decrease as histopathological grade increased (low grade = 1.471 ± 0.202 × 10^−3^ mm^2^/s, high grade = 1.321 ± 0.143 × 10^−3^ mm^2^/s, *p* < 0.001). This trend was consistent with observations for D (low grade = 1.339 ± 0.201 × 10^−3^ mm^2^/s, high grade = 1.129 ± 0.146 × 10^−3^ mm^2^/s, *p* < 0.001), D_app_ (low grade = 1.499 ± 0.231 × 10^−3^ mm^2^/s, high grade = 1.297 ± 0.224 × 10^−3^ mm^2^/s, *p* < 0.001), and f (low grade = 0.239 ± 0.068, high grade = 0.197 ± 0.055, *p* = 0.001). However, the behavior of K_app_ was contrary to this pattern (low grade = 0.678 ± 0.133, high grade = 0.819 ± 0.130, *p* < 0.001). Interestingly, IVIM-derived D_p_ did not exhibit a significant difference between low-grade and high-grade rectal cancer (low grade = 45.952 ± 12.376 × 10^−3^ mm^2^/s, high grade = 48.165 ± 12.368 × 10^−3^ mm^2^/s, *p* = 0.427).

### 3.3. The Correlations among the Different DWI-Derived Biological Markers

**Figure 6:**
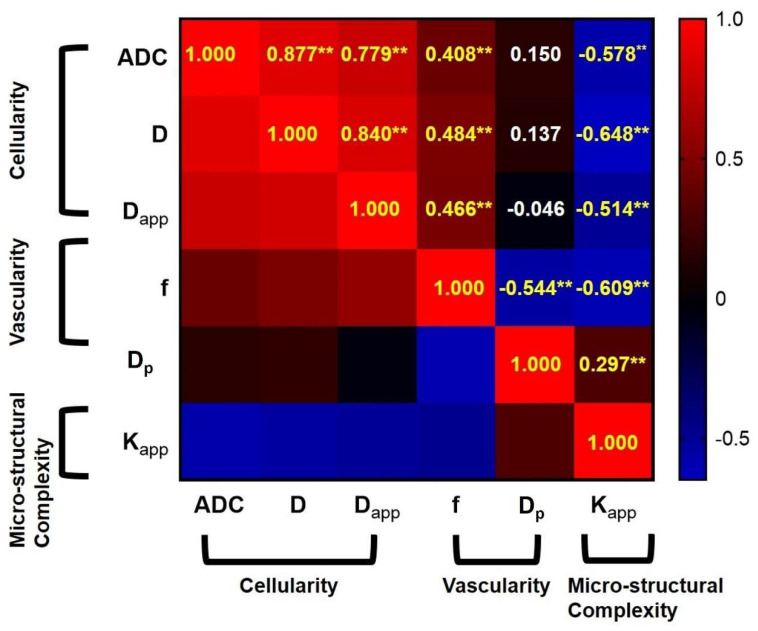
Correlation matrix showing the correlations among different DWI-derived biological markers. Note: (1) ** signifies *p* values of less than 0.01, (2) The values in the matrix represent the correlation coefficient abbreviated as r.

The correlation matrix in Figure 6 showed the correlations among various DWI-derived parameters. Having similar biological inspiration of cellular density, ADC, D, and D_app_ showed a strong positive correlation (r_ADC & D_ = 0.877, *p* < 0.01; r_ADC & Dapp_ = 0.779; *p* < 0.01, r_D & Dapp_ = 0.840, *p* < 0.01). In addition, cellular density related ADC, D and D_app_ significantly and positively correlated with the vascularity related f (r_ADC & f_ = 0.408, *p* < 0.01; r_D & f_ = 0.484, *p* < 0.01; r_Dapp & f_ = 0.466, *p* < 0.01), but significantly and negatively correlated with structural heterogeneity related K_app_ (r_ADC & Kapp_ = −0.578, *p* < 0.01; r_D & Kapp_ = −0.648, *p* < 0.01; r_Dapp & Kapp_ = −0.514, *p* < 0.01). Moreover, there were significant negative correlations between f and D_p_ (r_f & Dp_ = −0.544, *p* < 0.01), as well as f and K_app_ (r_f & Kapp_ = −0.609, *p* < 0.01) with regard to significant positive correlation between D_p_ and K_app_ (r_Dp & Kapp_ = 0.297, *p* < 0.01).

### 3.4. Diagnostic Performance Evaluation

**Figure 7:**
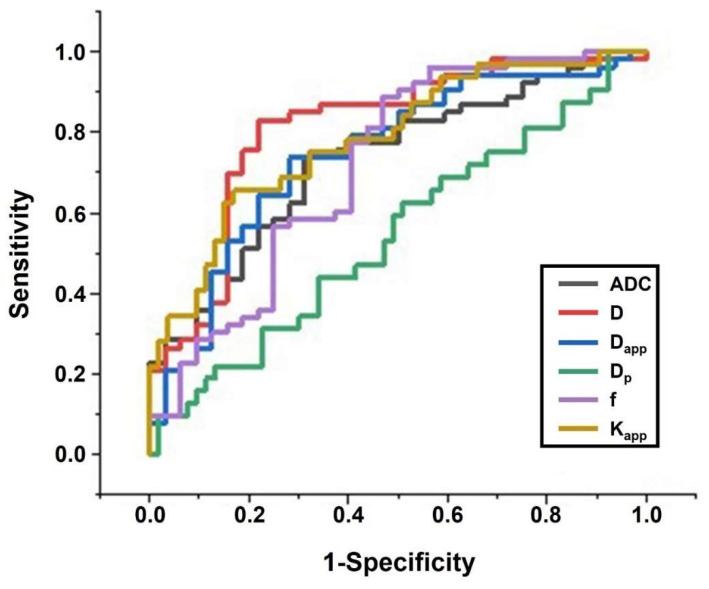
Diagnostic power of individual DWI-derived parameters.

**Figure 8:**
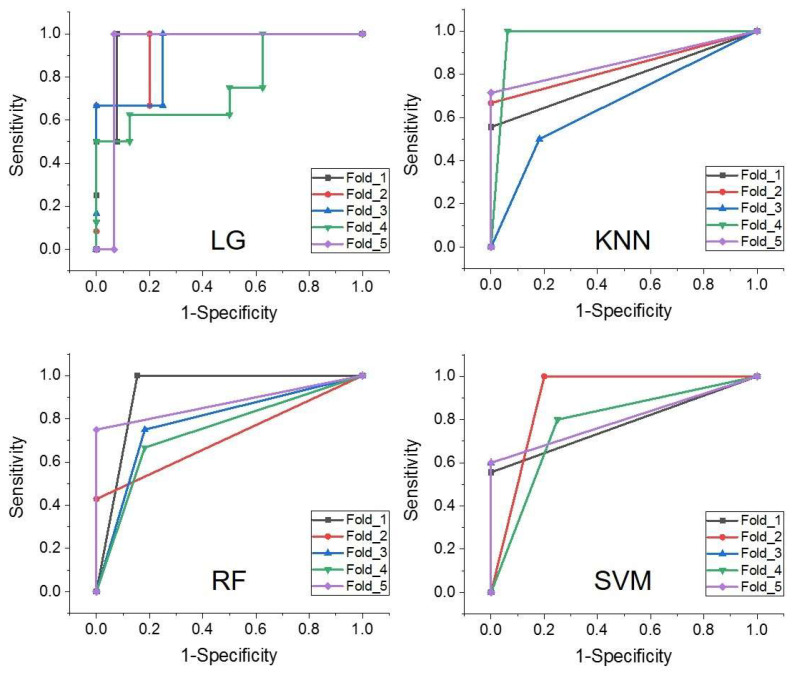
ROC analysis with 5-fold cross validation of four machine learning-based models.

Figure 7 and Table 3 present the comparisons of the diagnostic efficacy of individual DWI-derived parameters for grading rectal cancer. Among the individual DWI-derived parameters, the parameter associated with cellularity, D, demonstrated the highest grading power (AUC = 0.811, 95% confidence interval (CI): 0.711–0.911), followed by K_app_ (AUC = 0.782, 95% CI: 0.681–0.884), D_app_ (AUC = 0.746, 95% CI: 0.635–0.856), ADC (AUC = 0.729, 95% CI: 0.620–0.838), f (AUC = 0.718, 95% CI: 0.598–0.838), and D_p_ (AUC = 0.543, 95% CI: 0.415–0.671). It is noteworthy that for four machine learning-based diagnostic models, we introduced cellularity-related D, vascularity-related f, and heterogeneity-related K_app_ as input features. The rationale for selecting these parameters is as follows: These parameters exhibited significant differences between low-grade and high-grade rectal cancer. They provide distinct biological insights, encompassing cellularity (D), vascularity (f), and micro-structural complexity (K_app_). D_p_ was excluded from consideration because it showed no significant difference between low-grade and high-grade rectal cancer, and D_app_ was omitted due to the following reasons: (1) D_app_ conveys the same biological insight as D. (2) By eliminating the influence of perfusion, D is better suited to characterize the true diffusion restriction arising from cellularity [24,27]. The diagnostic performance of the four machine learning-based classifiers was evaluated through 5-fold cross-validation and is depicted in Figure 8 and Table 3. Specifically, LG exhibited the most potent grading power (AUC in 5 folds: 0.962, 0.933, 0.917, 0.766, 0.933. Mean AUC: 0.902, 95% CI: 0.754–1.000), followed by KNN (AUC in 5 folds: 0.778, 0.833, 0.659, 0.969, 0.857. Mean AUC: 0.819, 95% CI: 0.590–0.964), SVM (AUC in 5 folds: 0.778, 0.900, 0.800, 0.775, 0.800. Mean AUC: 0.811, 95% CI: 0.628–0.982), and RF (AUC in 5 folds: 0.923, 0.714, 0.784, 0.742, 0.875. Mean AUC: 0.808, 95% CI: 0.628–0.975). Additional detailed measures of diagnostic power, including sensitivity, specificity, and Youden Indexes, are presented in Table 3. Furthermore, a Delong test was conducted to compare the AUC of the best machine-learning classifier (LG-based diagnostic models) with the AUCs of other individual DWI-derived parameters. The results indicated that the AUC of the LG-based diagnostic model was significantly higher than the AUCs of any other individual DWI parameters (*p* < 0.05).

## 4. Discussion

In recent years, DWI-MRI has garnered significant interest among radiologists due to its capacity to provide various biological markers closely linked to disease processes, particularly carcinogenesis. With the aid of diverse DWI models that have emerged in recent times, DWI-derived insights, encompassing aspects such as cellularity, vascularity, and micro-structural heterogeneity, have demonstrated substantial clinical promise. For instance, commonly used DWI-derived parameters such as ADC, DKI-derived D_app_, and IVIM-derived D, all of which are capable of representing cellular density, exhibit a notable decrease as tumor malignancy escalates [8,9,28]. Indeed, parameters like DKI-derived K_app_, which provide insights into structural heterogeneity, demonstrate notable variations as tumor malignancy increases [8,29,30]. Furthermore, it has been previously reported that IVIM-derived f possesses diagnostic potential for grading rectal cancer, as it allows for the quantification of changes in micro-circulation [22]. Our findings align with these earlier studies and corroborate the work of Zhu et al. and Cui et al., who concluded that the D value decreases while K_app_ increases with an increase in the histopathologic grade of rectal cancer [7,8]. Additionally, Sun et al. found that decreased IVIM-derived D and f values hold substantial diagnostic power for grading rectal cancer. The biological underpinnings of these findings can be elucidated as follows: (1) As the histopathological grade of rectal cancer rises, the rapid proliferation of tumor cells results in a marked increase in the ratio of nuclear to cytoplasmic content. This, in turn, restricts the diffusion of water molecules, leading to a decrease in ADC, D, and D_app_ values. (2) K_app_ is defined as a measure of deviation from a perfect DWI Gaussian distribution. Increased tissue heterogeneity associated with higher histopathologic grade results in a greater deviation from this Gaussian distribution, leading to a significant increase in K_app_. (3) High-grade rectal cancer is characterized by the rapid growth of tumor cells, leading to the poor structural integrity of lumenized vessels. This, in turn, diminishes micro-circulation and perfusion, ultimately reflected in a decrease in f value.

Regarding IVIM-derived D_p_, the lack of significant differences among different subgroups may be attributed to several factors. According to IVIM theory, D_p_ is influenced by various factors, as expressed by the equation: $D_{p}=(l\times v)/6$, where l represents capillary length, and v represents the average velocity of blood in the capillary [31]. Consequently, D_p_ is susceptible to the effects of a low signal-to-noise ratio (SNR). Moreover, many investigators have reported similar results, indicating that D_p_ does not exhibit significant differences among various subgroups [27,32,33].

The strong correlations observed among different DWI biological markers underscore the importance of integrating these insights to comprehensively characterize tumors from various angles, considering cellular density, vascularity, and micro-structural heterogeneity. While combinations of different DWI models have been widely explored in the literature, few studies have focused on integrating these models based on DWI-derived biological insights. Much attention has been devoted to comparing the clinical effectiveness of different DWI parameters for specific clinical objectives such as grading and staging. However, an excessive number of DWI models can lead to prolonged scan times and complex procedures, potentially diminishing their clinical value. In this research, we believe that by combining mono-exponential DWI, DKI, and IVIM, we can effectively capture the key biological insights related to cellularity, vascularity, and heterogeneity. Therefore, we employed multiple machine learning classifiers to integrate these different DWI-derived biological insights for preoperative grading of rectal cancer. Prior attempts have been made to apply machine learning to enhance the clinical effectiveness of DWI. For instance, Wang achieved accurate diagnosis of prostate cancer (AUC = 0.983) by applying DKI-derived radiomics metrics and an SVM-based classifier [34]. Vidic et al. suggested that integrating IVIM-derived histogram metrics with SVM effectively assisted in discriminating malignant breast cancer from benign breast cancer with an accuracy of 0.98 [35]. In a different approach, Yin Bo’s research used three different machine learning classifiers, including classic decision trees, conditional inference trees, and decision forests, to demonstrate that ADC map-based texture features had significant potential for accurately grading meningioma [36]. However, to the best of our knowledge: (1) Combining multiple DWI models to yield multiple biological insights for use as input features in machine learning models has been rarely explored. (2) Few studies have introduced and compared multiple machine learning models instead of relying on a single classifier like SVM. (3) The integration of machine learning with DWI for preoperative grading of rectal cancer has been underexplored. As anticipated, machine learning classifiers based on logistic regression demonstrated superior diagnostic performance for grading rectal cancer compared to using single DWI-derived parameters. This improvement can be attributed to several factors: (1) The simultaneous introduction of three DWI biological markers allowed for a more comprehensive characterization of rectal cancer. (2) Machine learning algorithms inherently possess robust classification abilities. The logistic regression model is computationally efficient, easy to implement, and requires less time and computer memory. It is also highly robust to data noise.

Nonetheless, it is important to acknowledge several potential limitations in this study. Firstly, akin to the majority of previous research, the positive results—which demonstrate that DWI-derived parameters can offer substantial diagnostic power (for grading, staging, or therapeutic efficacy evaluation) across various cancers—merely suggest the clinical potential of DWI. The practical application of these DWI-derived parameters in clinical settings may still be a long-term goal. The challenges stemming from unstable measurements or parameter calculations, induced by the inherent limitations of DWI-MRI, including the low SNR of DWI images and issues like distortion or mis-registrations, particularly at high b values, are challenges faced by nearly all investigators. Addressing these issues will require collaborative efforts from researchers in the future. Secondly, the sample size and data imbalance may introduce certain complexities during the training of machine learning models. Thirdly, the inclusion of specific pathological indices as input features could potentially enhance the diagnostic power of the models. Fourthly, in future studies, the utilization of more robust deep learning models may further benefit the construction of diagnostic models.

## 5. Conclusions

The combination of different DWI techniques allows for the comprehensive characterization of rectal cancer from multiple perspectives, encompassing aspects such as cellularity, vascularity, and micro-structural heterogeneity. Integrating these DWI-derived biological markers with multiple machine learning classifiers, we can achieve exceptional diagnostic performance for grading rectal cancer. This approach, which relies on multiple DWI models, multiple DWI-derived biological markers, and various classifiers, holds significant promise for accurately preoperatively grading rectal cancer. Furthermore, this diagnostic strategy can be readily extended to other clinical applications, including cancer diagnosis, cancer staging, and the prediction of therapeutic outcomes.

## Figures and Tables

**Table 1:** Baseline clinical-pathological characteristics.

Characteristics	Low Grade (n = 53)	High Grade (n = 32)	*p* Value
Age: mean ± SD (years)	59.13 ± 10.75	61.27 ± 11.54	0.612
Gender			0.703
Men	28	17	
Women	25	15	
Size of tumor (mm)	24.1 ± 8.79	38.3 ± 9.25	0.085
pT stage			0.047
T1	3	2	
T2	28	7	
T3	15	13	
T4	7	10	
pN stage			0.452
N0	20	15	
N1	17	10	
N2	16	7	
CA199			0.251
≤20 U/mL	36	13	
>20 U/mL	17	19	
CEA			0.632
≤5 ng/mL	30	10	
>5 ng/mL	23	22	

**Table 2:** The inter-observer agreement of DWI-derived parameters as measured by three radiologists.

	ICC	Lower Bound of 95% CI	Upper Bound of 95% CI
ADC	0.862	0.821	0.883
D	0.827	0.787	0.840
D_p_	0.751	0.734	0.792
f	0.802	0.761	0.829
D_app_	0.836	0.801	0.855
K_app_	0.843	0.817	0.862

**Table 3:** Evaluation of the diagnostic performance of individual DWI-derived parameters and machine learning-based diagnostic models.

	Sensitivity	Specificity	AUC	95% CI of AUC	Youden Index
KNN	0.687	0.951	0.819	0.590–0.964	0.638
LG	0.925	0.856	0.902	0.754–1.000	0.781
RF	0.719	0.897	0.808	0.628–0.975	0.616
SVM	0.711	0.910	0.811	0.628–0.982	0.621
ADC	0.686	0.736	0.729	0.620–0.838	0.423
D	0.781	0.830	0.811	0.711–0.911	0.611
K_app_	0.656	0.830	0.782	0.681–0.884	0.486
D_app_	0.719	0.736	0.746	0.635–0.856	0.455
f	0.531	0.887	0.718	0.598–0.838	0.418
D_p_	0.625	0.490	0.543	0.415–0.671	0.116

KNN: K nearest neighbor, LG: logistic regression, RF: random forest, SVM: support vector machine, ADC: apparent diffusion coefficient (mono-exponential DWI), D: true diffusion coefficient, D_p_: pseudo diffusion coefficient, K_app_: apparent kurtosis coefficient, D_app_: apparent diffusion coefficient (DKI), f: perfusion fraction, D_p_: pseudo diffusion coefficient and CI: confidence interval.

## Data Availability

Owing to ethical considerations and the importance of safeguarding patient privacy, medical DICOM data cannot be made available. Nevertheless, access to other data and results can be arranged upon request from the corresponding author, under reasonable conditions.
